# Programmable deformation of patterned bimorph actuator swarm

**DOI:** 10.1093/nsr/nwz219

**Published:** 2020-01-20

**Authors:** Jia-Nan Ma, Yong-Lai Zhang, Dong-Dong Han, Jiang-Wei Mao, Zhao-Di Chen, Hong-Bo Sun

**Affiliations:** 1 State Key Laboratory of Integrated Optoelectronics, College of Electronic Science and Engineering, Jilin University, Changchun 130012, China; 2 State Key Laboratory of Precision Measurement Technology and Instruments, Department of Precision Instrument, Tsinghua University, Beijing 100084, China

**Keywords:** graphene oxide, actuators, moisture responsive, predictable deformation, soft robots

## Abstract

Graphene-based actuators featuring fast and reversible deformation under various external stimuli are promising for soft robotics. However, these bimorph actuators are incapable of complex and programmable 3D deformation, which limits their practical application. Here, inspired from the collective coupling and coordination of living cells, we fabricated a moisture-responsive graphene actuator swarm that has programmable shape-changing capability by programming the SU-8 patterns underneath. To get better control over the deformation, we fabricated SU-8 micropattern arrays with specific geometries and orientations on a continuous graphene oxide film, forming a swarm of bimorph actuators. In this way, predictable and complex deformations, including bending, twisting, coiling, asymmetric bending, 3D folding, and combinations of these, have been achieved due to the collective coupling and coordination of the actuator swarm. This work proposes a new way to program the deformation of bilayer actuators, expanding the capabilities of existing bimorph actuators for applications in various smart devices.

## INTRODUCTION

Actuators that can convert various environmental stimuli to mechanical works have revealed great potential for developing smart devices such as soft robots [1,2], micro-electromechanical systems (MEMS) [3,4] and automatic Lab-on-a-Chip systems [5,6]. Generally, actuators can be manipulated by various external stimuli such as humidity [7,8], light [9–13], pH value [14], heat [15], chemicals [16], magnetic/electric fields [17,18] and the combination of two or more of them. To realize revisable and controllable deformation, bilayer structure has been widely used for design and fabrication of stimuli-responsive actuators owing to their unique advantages of sensitive response, large-scale deformation and ease of preparation [19,20]. A general concept for developing bimorph actuators is to construct an active layer and an inert layer by simple deposition [21], filtration [20], electrospinning [22] and spin coating [23]. Under external stimuli, the active layer may swell or shrink, whereas the inert layer would remain unchanged. In this way, the mismatch in strain at the bilayer interface directly causes bending or twisting of the bilayer. In the past decade, to pursue fast and large-scale deformation, great efforts have been devoted to the development of new stimuli response materials (SRMs).

To date, various SRMs have been successfully developed and employed for bimorph actuators. For instance, hydrogel that is very sensitive to environmental humidity has been used for moisture-responsive actuators [24]; photothermal polymers that can convert light to heat effectively enable light actuation [25–27]; electrothermal materials can realize controllable deformation through electric–thermal conversion [28]; phase-change materials and photoisomerization effects are promising for actuator design [29]. As typical examples, Sun *et al.* prepared energetic moisture-responsive actuators comprising poly(acrylic acid)/poly(allylamine hydrochloride) hydrogels and UV-cured Norland Optical Adhesive 63, in which the moisture changes induced the large mismatch in hygroscopic expansion of the two materials, and led to the bending behavior of the bilayer [30]. Zhang *et al.* reported light-responsive smart curtains and motors based on a polycarbonate/single-walled carbon nanotube double-layer structure, realizing bending deformation [20]. Zhu *et al.* introduced soft electrothermal actuators with a polyimide (PI)/silver nanowire/polydimethylsiloxane (PDMS) sandwich structure, which could bend towards the PI side under electrical actuation [31]. Liu *et al.* prepared an intelligent Cr/VO_2_ bi-layer claw with a size of ∼100 μm using a deposition and etching method, and drove the claw via temperature change-induced phase transition of VO_2_ [32].

Recently, graphene and graphene oxide (GO) that possess a series of outstanding properties such as high electrical/thermal conductivity, good mechanical flexibility, excellent biocompatibility and good stability have emerged as a new type of smart material for actuator design [33–38]. Typically, Qu *et al.* successfully performed surface plasma treatment of hexane and oxygen on the positive and negative sides of the reduced graphene oxide, producing an electrochemically driven smart actuator [39]. In our previous work, we fabricated graphene-based moisture-responsive actuators using a self-controlled photoreduction method, demonstrating the moisture-induced bending and twisting [40]. Despite the fact that rapid progress has been made in this dynamic field, current development of bimorph actuators mainly depends on the advancement of novel SRMs. Less attention has been paid to the refined control of their deformation. At present, graphene-based bilayer actuators are only capable of simple deformation, such as bending. Despite some previous works having proved the possibility of bending direction control by producing a patterned constrained layer [41,42], their deformation is passively restricted due to the anisotropic mechanical resistance. Currently, the development of bimorph actuators that enable active and programmable deformation remains a challenging task.

In this work, inspired from the collective coupling and coordination of living cells, we developed a self-healing graphene actuator swarm that enables programmable 3D deformation by integrating SU-8 pattern arrays with GO. The SU-8 micropattern arrays with specific geometries and orientations can couple with the GO film, forming a swarm of bimorph actuators in which an individual SU-8/GO bilayer can serve as an actuator ‘cell’. Therefore, by controlling the size, shape and orientation of the SU-8 patterns, more complex deformations can be programmed due to the collective coupling and coordination of the actuator swarm. The present method may open up a new way for precisely controlling the deformation of graphene-based actuators.

## RESULTS AND DISCUSSION

**Figure 1.**
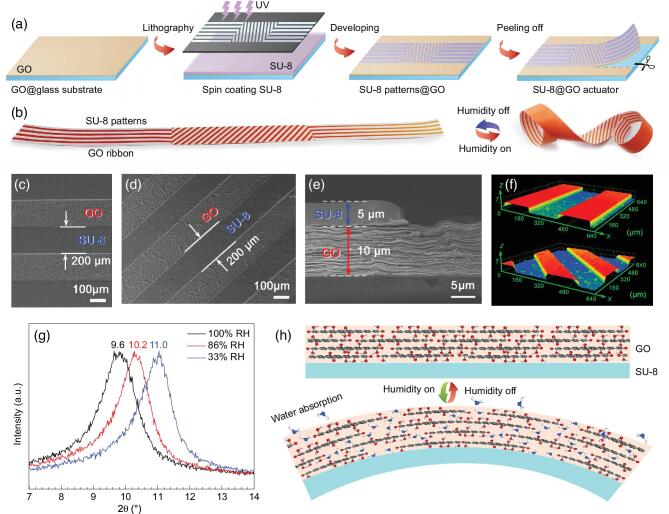
Design principle and the fabrication procedure of moisture-responsive SU-8/GO bimorph actuator swarms. (a) Schematic illustration of the fabrication of patterned SU-8/GO bilayer film using UV lithography. (b) The paper model of patterned SU-8/GO ribbon and its predictable moisture-responsive deformation under humidity actuation. (c, d) Surface SEM images of patterned SU-8/GO. (e) Cross-section SEM image of patterned SU-8/GO bilayer structure. (f) CLSM images of the patterned SU-8/GO bilayer structure. (g) XRD patterns of GO paper under different RHs. (h) Schematic illustration of the interaction between water molecules and GO nanosheets in dry air and moisture.

Figure 1a schematically illustrates the fabrication process of the patterned SU-8 and GO bimorph actuator. As a moisture-active layer, GO solution (5 mg/mL) was dripped on the clean glass substrate and dried at room temperature. Considering the poor water adsorption property, rigidity and the ease of patterning, SU-8 has been coupled with GO as a patterned moisture-inert layer. The SU-8 photopolymer was spin-coated on the top of GO film and patterned by UV lithography. After the developing process, the patterned bilayer was peeled off from the glass substrate and cut into strips. The model shown on the left of Fig. 1b demonstrates a typical SU-8 and GO bilayer ribbon. It can deform into a complex shape under humidity stimulation (right of Fig. 1b) and return to its original shape when the humidity is off. Scanning electron microscopy (SEM) images (Fig. 1c and d) of the SU-8/GO bilayer show the uniform, periodic SU-8 stripes with the width and spacing of ∼200 μm on the GO surface. Additionally, the cross-section SEM image (Fig. 1e) demonstrates the layered structure of GO (∼10 μm in thickness) and the bulk structure of SU-8 (∼5 μm in thickness) with good interlayer contact. Furthermore, the confocal laser scanning microscopy (CLSM) stereograms obtained using a confocal microscope (Fig. 1f) show the morphology of SU-8/GO ribbon from a 3D perspective, indicating the patterned constrained layer of photopolymer SU-8. Here, it is necessary to point out that SU-8 is not the only choice for this kind of actuator, as a moisture-inert layer, other hydrophobic photopolymers that permit flexible patterning are also workable.

The moisture-responsive mechanism of the patterned SU-8/GO bilayer has been investigated by analyzing the chemical composition of GO. Typically, X-ray photoelectron spectroscopy (XPS) is a common method to quantitatively investigate the chemical composition of GO. As shown in Supplementary Fig. S1a, the C1s XPS spectrum of GO shows three peaks: C–C (non-oxygenated ring carbon) at 284.7 eV, C–O (hydroxyl and epoxy carbon) at 286.8 eV, and C=O (carbonyl) at 288.1 eV, respectively. The proportion of C–O and C=O is as high as 57%, indicating that oxygen-containing groups (OCGs) are very rich in GO. The survey spectrum of GO shows a more obvious comparison (Supplementary Fig. S1b), the carbon to oxygen atom ratio is ∼2, which confirms the presence of a large amount of OCGs. Additionally, the Fourier transform infrared (FTIR) spectrum (Supplementary Fig. S1c) of GO film is in good agreement with the XPS results. GO shows a set of transmission bands corresponding to OCGs (e.g. the strong C=O peak at 1731 cm^−1^, the bending vibration of O–H at 1373 cm^−1^, the stretching vibration of C–OH at 1246 cm^−1^ and the stretching vibration of C–O in epoxy groups at 1080 cm^−1^). Since GO contains abundant hydrophilic OCGs, it can adsorb a large amount of water molecules because of the formation of hydrogen bonds under moisture conditions. Thermogravimetric (TG) analysis was also conducted under air atmosphere to evaluate the thermal stability and the mass loss upon thermal reduction (Supplementary Fig. S1d). A weight loss of ∼13% at ∼118°C has been observed due to desorption of water, and a rapid weight loss of ∼29% from 160 to 250°C was detected due to the removal of OCGs. To quantitatively determine the structural change upon water

adsorption, we used X-ray diffraction (XRD) to measure the interlayer spacing changes of GO nanosheets under different relative humidity (RH). As shown in Fig. 1g, at the RH of 33%, GO film showed a diffraction peak at ∼11.0°, corresponding to a *d*-spacing of ∼0.8 nm. When the humidity was increased to 86% and 100% RH, the diffraction peaks shifted to 10.2° and 9.6°, respectively, indicating the increase of interlayer *d*-spacing. At 100% RH, the *d*-spacing is determined to be ∼0.92 nm, which indicates an increase of 15% compared to that at 33% RH. Based on the above-mentioned experimental results, it is reasonable to deduce that a GO film can absorb abundant water molecules and then swell in both normal and in-plane directions. However, it is well known that the photopolymer of SU-8, mainly epoxy resin, is inert in moisture. Therefore, the difference in volume change under moisture actuation leads to a strain mismatch at the SU-8/GO interface, inducing the bending of the bilayer towards the SU-8 side (Fig. 1h).

**Figure 2.**
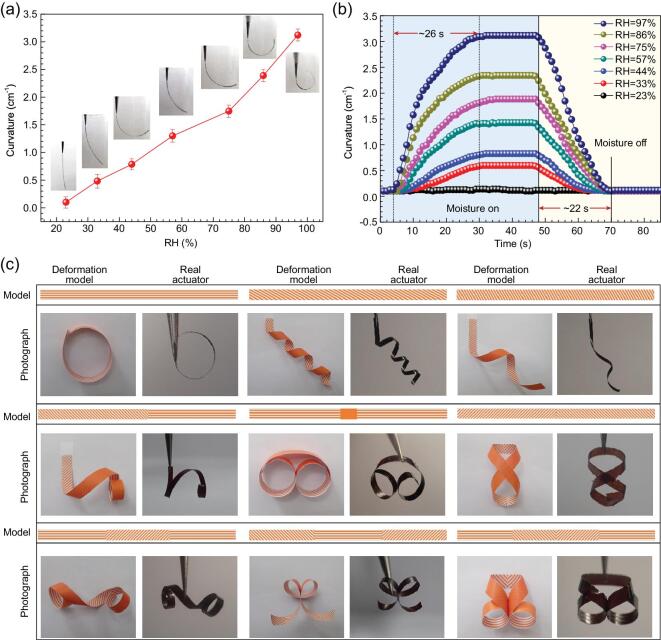
Moisture-responsive property and moisture-induced complex deformations. (a) Dependence of the maximum bending curvature of the SU-8/GO bilayer paper on different RH. (b) Responsive and recovery properties of the SU-8/GO bilayer under different RH. (c) Complex deformations of different patterned SU-8/GO bilayers. Nine examples have been shown in this figure. In each example, the top image is the pattern model for SU-8 layer, the bottom left is the photograph of deformed paper model, and the bottom right is the photograph of the deformed real actuator of SU-8/GO bilayers. The dimensions of the actuator are 30 mm × 1.5 mm in size (length × width) and the RH is 100%.

To quantify the deformation behavior of the SU-8/GO bilayer film, we measured the bending curvature of an SU-8/GO ribbon (length: 20 mm; width: 1 mm) under different RH conditions (Fig. 2a). It can be clearly seen that the maximum bending curvature of the as-prepared SU-8/GO ribbon increased from 0.10 to 3.12 with the increase of RH from 23% to 97%. The insets of Fig. 2a display photos of the bending SU-8/GO ribbon when exposed to different RH conditions. To quantify the response sensitivity of the bilayer actuator, we define the sensitivity (*S*) as:

$$\begin{equation*}
S = \Delta C/\Delta {\rm{RH}},
\end{equation*}$$

where Δ*C* is the curvature change of the bilayer, and ΔRH is the change of relative humidity. In our work, the humidity-responsive sensitivity of the bilayer is calculated to be 0.041 cm^−1^**·** %RH^−1^, which is higher than other moisture-responsive bimorph actuators reported elsewhere (Supplementary Table S1). The bending–straightening deformation of the SU-8/GO bilayer ribbon is reversible. Since GO permits ultrafast permeation of water molecules [43], the response and recovery processes are very fast (Fig. 2b). The average response and recovery times are measured to be 26 s and 22 s, respectively. It is worth pointing out that the thicknesses of both SU-8 and GO layers can affect the deformation properties. When the thickness of SU-8 is fixed at 5 μm, with the increase of GO thickness from 8.5 to 11.5 μm, the bending curvature of the bilayer increases from 2.67 to 3.48 cm^−1^ (Supplementary Fig. S2). In this regard, to promote the deformation degree under moisture actuation, the GO layer should be thick enough to induce more obvious volume change. When the thickness of GO is fixed at 10 μm, the increase of SU-8 thickness shows a negative influence on the deformation degree due to the increase of resistance (Supplementary Fig. S3). In this work, considering the mechanical strength for self-supporting and the deformation degree for moisture actuation, we optimized the thickness of GO and SU-8 to be 10 μm and 5 μm, respectively.

Since the geometry, distribution and orientation of the SU-8 patterns can be flexibly designed, deformation of our bilayer can be freely controlled, which provides the possibility for programming their complex reconfiguration beyond simple bending. We first tuned the orientations of the SU-8 stripes. As shown in Fig. 2c, a variety of complex and predictable deformations have been achieved by patterning the SU-8 layer into stripes of different orientations. In the nine actuators, predictable and complex deformations, including bending, twisting, coiling and the combination of two or more deformations, have been demonstrated. Also, the patterned SU-8/GO bimorph actuator shows good stability with respect to surface morphology and high durability in 500 moisture actuation cycles (Supplementary Fig. S4). The patterned structure almost kept the same morphology after 500 times of bending without detectable swelling or deformation. Moreover, the interface between SU-8 and GO shows tight contact before and after frequent actuation (500 times), indicating the good interlayer adhesion (Supplementary Fig. S5).

**Figure 3.**
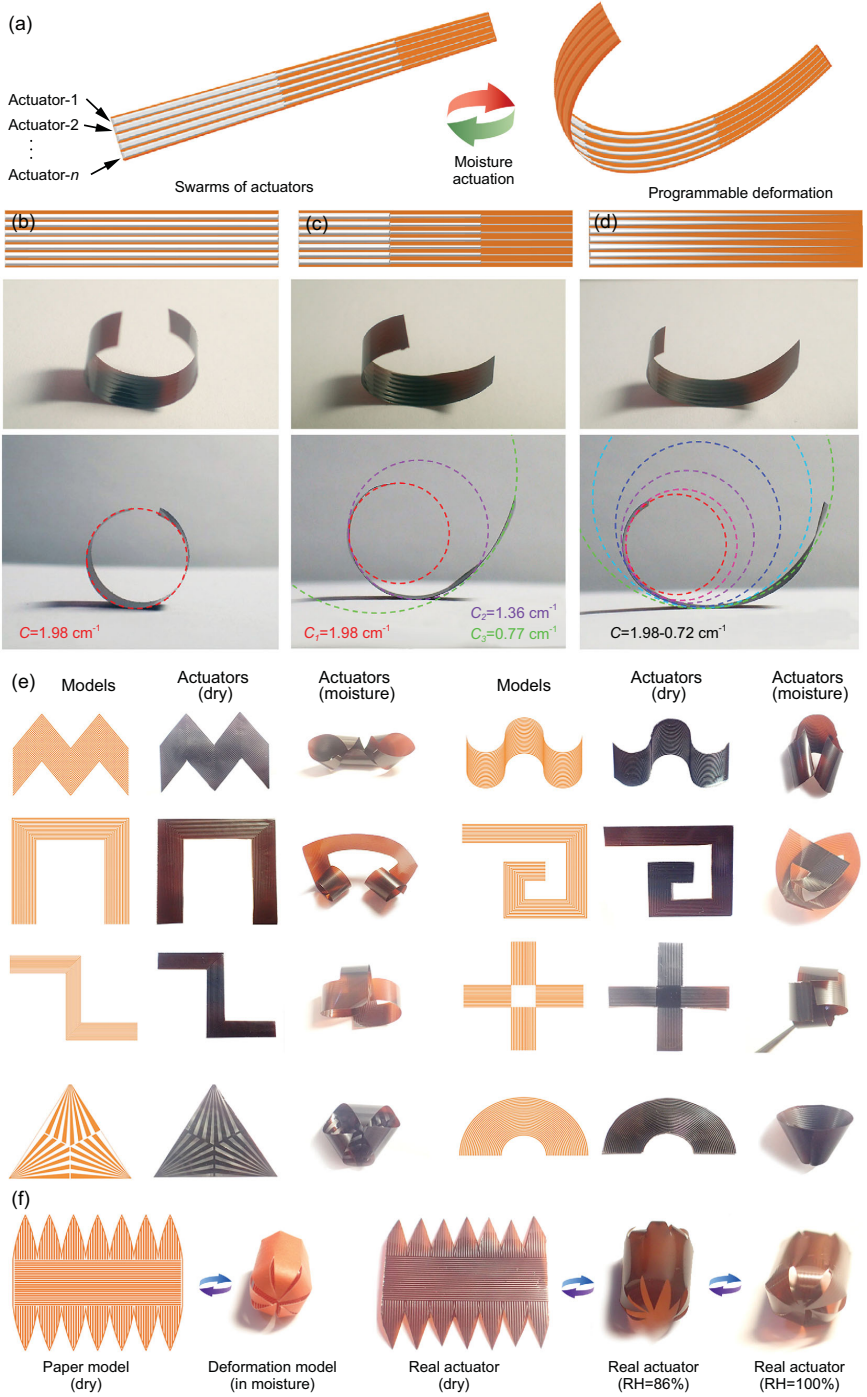
The basic concept of a bimorph actuator swarm and the programmable 3D deformations. (a) Schematic illustration of the design principle of swarms of actuators enabling programmable deformation. (b) The actuator based on periodic SU-8 stripes; a curvature of 1.98 cm^−1^ can be achieved under moisture actuation. (c) The actuator based on tri-block SU-8 patterns; the bending curvature for the tri-blocks is 1.98, 1.36 and 0.77 cm^−1^, respectively. (d) The actuator based on gradually narrowed SU-8 patterns; the curvature changes gradually from 1.98 to 0.72 cm^−1^. (b–d) From top to bottom are models, the photographs of the bending SU-8/GO bilayer ribbon and the side view of the curvature. The dimensions of the actuator are 30 mm × 4.7 mm in size (length × width) and the RH is 75%. (e) A series of complex 3D deformations of different SU-8/GO bilayers; from left to right are models, photographs of the real actuators under dry conditions and their deformation under moisture actuation. (f) The 3D capsule-shape deformation of a SU-8/GO actuator. The driving relative humidity is 100% RH.

Unlike those actuators that control their bending direction on the basis of anisotropic mechanical resistance, we realize programmable deformation through the collective coupling of actuator swarms. The design principle of our actuator swarm is shown in Fig. 3a. The SU-8 pattern arrays can be fabricated into any desired shapes, in which an individual SU-8 pattern is not connected with each other. In combination with the bottom GO layer, each SU-8 structure can form an individual bimorph actuator and deform actively under stimulation. In this regard, these SU-8/GO bilayer arrays can be considered as a swarm of actuators (actuator-1, actuator-2, … actuator-*n*). Under external stimulation, each actuator deforms individually, and the deformation of the entire structure is the collective coupling and coordination of the actuator swarm. To get better control over the moisture-triggered deformation, we further explored the size of the SU-8 patterns (e.g. width and spacing) on the humidity response properties. In our experiments, we first set the duty ratio (width/spacing of individual SU-8/GO actuators) at 1.0, and evaluated the influence of periods on the bending performance. As shown in Supplementary Fig. S6, we tuned the periods from 50 to 1000 μm, and the bending curvature increases and finally tends to saturation. Then, we fixed the period at 400 μm, and varied the duty ratio from 0.25 to 4.0 (Supplementary Fig. S7). With the increase of duty ratio, the bending curvature increased. The relationship between the pattern structure (e.g. period and duty ratio) against the bending curvature can be formulated based on a quadratic function fitting (Supplementary Figs S6 and S7). Since the deformation of the entire film is largely governed by the SU-8 patterns, the relationship function might be very helpful for designing more complex deformation of the bilayer film. In addition to the bending degree, the bending curvature at different position of a bilayer ribbon can be precisely tailored by programming the geometries of SU-8 patterns (Fig. 3b–d). For instance, using a tri-block and a triangle SU-8 array, asymmetric deformation such as a tri-block bending with three different curvatures and a gradually changed bending curvature can be achieved, respectively (Fig. 3b–d). In the case of uniform SU-8 stripes with the width of 500 μm and the space of 200 μm (Fig. 3b), a curvature of 1.98 cm^−1^ can be achieved under moisture actuation. Interestingly, when the SU-8 stripes are patterned into a tri-block array with the width of 500 μm, 300 μm and 100 μm, respectively, three different bending curvatures of 1.98 cm^−1^, 1.36 cm^−1^ and 0.77 cm^−1^ appear from left to right, respectively (Fig. 3c). These results confirm the possibility of programmable bending curvature control. Actually, the width and space can be continuously tailored. We employed a triangle SU-8 array in the actuator design (Fig. 3d), and it demonstrates a gradually changed bending curvature from 1.98 to 0.72 cm^−1^.

In our work, we find that the deformation of our bimorph actuator is governed by the collective coupling and coordination of the actuator swarm, in which the geometries and orientations of the SU-8 pattern are quite important for the bending direction and degree. For instance, we patterned parallel SU-8 stripes on a continuous GO film, which can be considered as a set of parallel and individual SU-8/GO bilayer actuator arrays (Supplementary Fig. S8a). Considering the extremely large aspect ratio of each SU-8/GO bilayer ribbon, it would prefer bending along the long side. To prove this hypothesis, we simulated the bending behavior of an individual SU-8/GO actuator with the help of finite element analysis software (supplementary Fig. S8b). In fact, for the rectangular ribbon of SU-8/GO, bending would not only occur along the long side, but also along the short side because of the isotropic expansion of GO. However, the degree of bending is largely different along the two directions. Supplementary Fig. S8c and d shows a view of the *X*–*Z*, *Y*–*Z* plane, respectively. It can be clearly seen that there is a larger curvature along the long side than that along the short side. According to the change of the corresponding coordinates before and after the humidification, we can calculate that the curvature along the long side is 0.249 based on the Pythagorean Theorem, whereas the curvature along the short side is only 0.029, which is an order of magnitude lower. In this regard, bending generally occurs along the long side of an individual SU-8/GO bilayer. And the deformation of the entire film is largely governed by the collective coupling effect of the actuator swarms.

To further extend the capability of more complex 3D deformation, we have fabricated more complex SU-8 patterns, in which periodic stripes with different geometries and non-linear orientations have been integrated together for controllable 3D reconfiguration. As shown in Fig. 3e, a series of complex 3D deformations, such as funnel-shape, asymmetric bending, complex 3D folding, and the arbitrary combination of them, have been demonstrated. Additionally, as a typical example of 3D deformation, a capsule-shaped actuator has been realized by precisely designing the patterns of SU-8 (Fig. 3f). Under moisture stimulation, the actuator gradually deforms into a 3D capsule shape from a 2D film. Such complex deformation indicates the potential of our actuator swarms.

**Figure 4.**
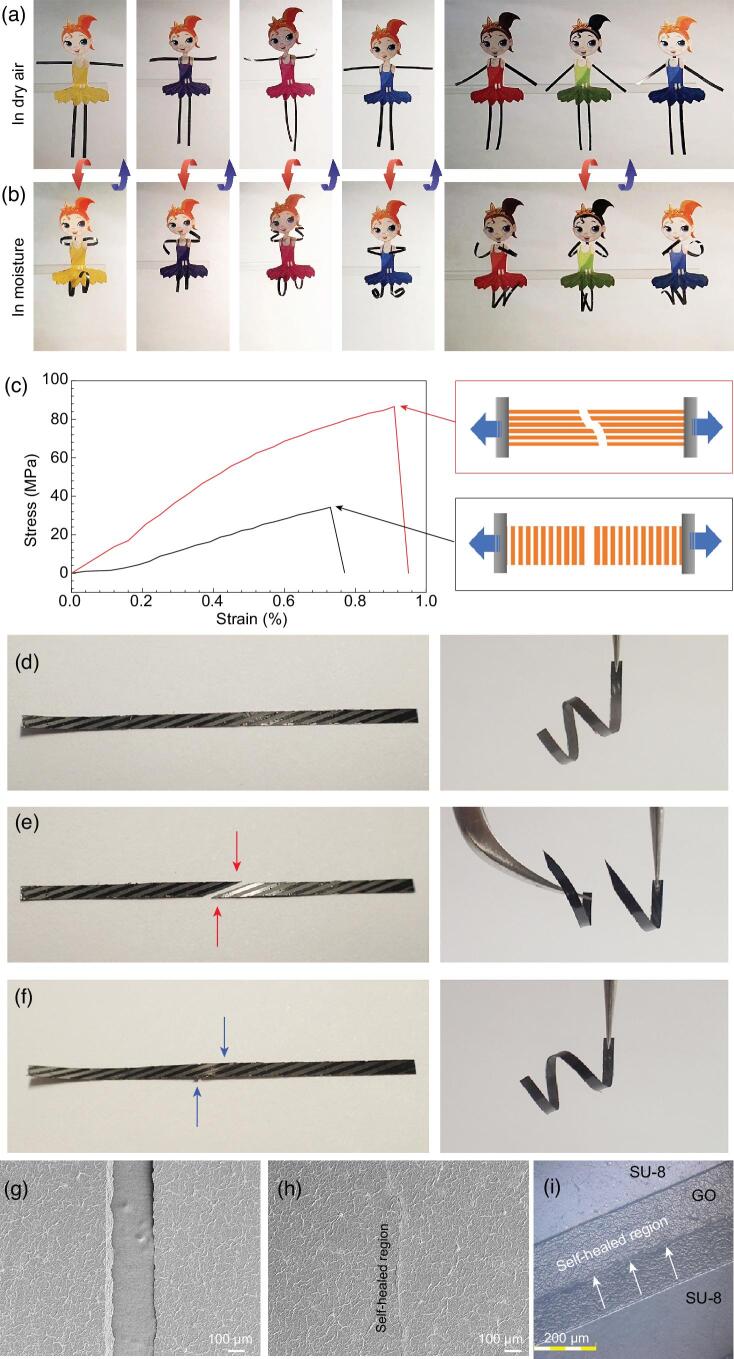
Dancer robots based on the bimorph actuator swarm and their self-healing properties. (a) The photograph of the dancers in dry air (initial state). (b) The ‘dancing’ state of dancers in moisture. The dimensions of the ‘smart arms and legs’ are 20 mm × 1.5 mm, 30 mm × 1.5 mm in size (length × width), respectively, and the RH is 100%. (c) Tensile tests of SU-8/GO ribbon with SU-8 stripes along and against the tensile direction. (d–f) The self-healing property of a SU-8/GO bi-layer ribbon. (d) The photographs of a SU-8/GO actuator in dry air (left) and moisture (right), respectively. (e) The photographs of a broken SU-8/GO actuator in dry air (left) and moisture (right), respectively. (f) The photographs of the self-healed SU-8/GO actuator in dry air (left) and moisture (right), respectively. (g) SEM image of the crack of the SU-8/GO actuator. (h) SEM image of the self-healed region. (i) CLSM image of the self-healed SU-8/GO actuator. The dimensions of the actuator are 30 mm × 1.5 mm in size (length × width), and the RH is 100%.

Taking advantage of the programmable deformation properties, soft robots can be fabricated through a designed manner. To demonstrate its full potential in robotics, we mimic the dancing process by integrating four patterned stripes as ‘smart arms and legs’ with a paper body. Since different patterns will lead to different deformations, we designed various patterns for these arms and legs. Under ambient humidity, these ‘dancer robots’ maintain a ‘standing’ state (Fig. 4a). When humidity is applied using a homemade moisture-supplying system (Supplementary Fig. S9), they start to dance following the designed action (Fig. 4b). The deformation of arms and legs are reversible, and they return to their original states when the humidity is switched off. By switching the humidity according to the melody of music, the ‘dancer robots’ may dance in time to music (Supplementary Movie 1).

Besides, to further highlight the significance of our swarm-type actuators, we also demonstrated the self-healing properties of our SU-8/GO actuators (Fig. 4c–i). Due to the presence of anisotropic SU-8 patterns, the mechanical strength parallel and perpendicular to the SU-8 stripes is different. As shown in Fig. 4c, the tensile strength of the actuator along the SU-8 stripes is much larger than that perpendicular to the SU-8 stripes. Thus, under external stretching, the bilayer tends to be broken at the GO gap between two neighboring SU-8 ribbons (right of Fig. 4c). To prove the self-healing properties, we investigated the moisture-responsive deformation of an SU-8/GO ribbon before and after self-healing. Notably, the pristine bilayer ribbon deforms obviously upon exposure to moisture (Fig. 4d). After dividing into two pieces, the as-form two actuators are also workable (Fig. 4e), because our actuators deform based on the swarm effect, and no damage has been done on each individual actuator. Interestingly, the cracks on the GO film (the gap between two individual stripes) demonstrated a self-healing property upon exposure to moisture, and the self-healed actuator can perform similarly to the pristine one (Fig. 4f). SEM images show the surface morphology change of the GO crack before and after self-healing (Fig. 4g and h), in which the GO crack has been well cured in moisture due to the re-assembly of GO sheets. The CLSM image (Fig. 4i) further confirms the recovery of the broken SU-8/GO ribbon, where only a minor healing trace can be detected. The self-healing property of our SU-8/GO actuators further suggests their robustness for practical applications in soft robots.

**Figure 5.**
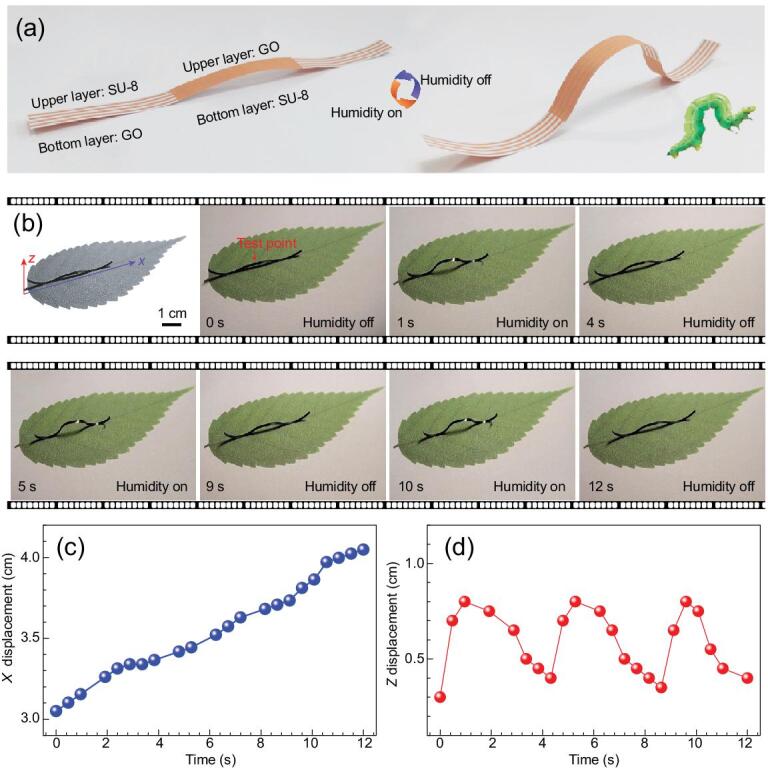
A simple caterpillar robot. (a) Schematic illustration of the biomimetic smart caterpillar model. (b) Moisture-responsive ‘smart caterpillar’ fabricated by patterned SU-8/GO. (c) Dependence of *X* displacement of the ‘smart caterpillar’ on time. (d) Dependence of *Z* displacement of the ‘smart caterpillar’ on time. The dimensions of the ‘smart caterpillar’ are 30 mm × 1.5 mm in size (length × width), and the RH is 100%.

Inspired from natural caterpillars that can move forward through the peristaltic movement, we designed a simple caterpillar robot using our SU-8/GO actuators. We divided the caterpillar robot into three parts: forelimb, body and hindlimb. Accordingly, we patterned the SU-8 constrained layer on both sides of a GO ribbon at the left, middle and right position, respectively, as shown in the model diagram of Fig. 5a. Under moisture actuation, the caterpillar will deform by bending the forelimb, body and hindlimb, just like an actual creeping caterpillar (inset of Fig. 5a). In our experiment, the smart caterpillar can move ∼1 cm by switching the local humidity three times (Fig. 5b and Supplementary Movie 2), in which humidity is controlled by the homemade moisture-supplying system (Supplementary Fig. S10). We further investigated the motion track by choosing the middle point as the test point. As shown in Fig. 5c, the creeping speed is uniform, and the displacement of the smart caterpillar along the *X* direction increases ∼1 cm within 12 s. We also tested the moving track along the *Z* direction. Since the movement is creeping, the moving track along the *Z* direction is periodic (Fig. 5d). Taking advantage of the patterned SU-8 constrained layer, the deformation of the bilayer can be well controlled. In this way, there is great potential to design and fabricate many types of robots beyond the examples shown above.

## CONCLUSION

In conclusion, a moisture-responsive graphene actuator swarm that can achieve complex and predictable deformation has been successfully prepared by coupling SU-8 patterns with GO film. The moisture-responsive mechanism and the responsive properties have been investigated. A series of complex deformations including bending, twisting, coiling and the combination of them have been achieved by making different SU-8 patterns. By tuning the geometries, distributions and the orientations of the SU-8 patterns, the actuator swarm enables programmable deformation due to the collective coupling and coordination effect. In addition, the SU-8/GO bimorph actuator swarm shows a moisture-triggered self-healing property, revealing robustness for practical usage. As a proof-of-concept, we further developed several moisture-responsive paper robots that can realize multiform deformations under moisture actuation, including smart ‘dancer robots’ that can dance with the humidity change and a smart ‘caterpillar robot’ mimicking the creep of a caterpillar. The moisture-responsive graphene actuators that can perform complex and predictable deformation may hold great promise for developing various smart graphene-based devices.

## METHODS

### Synthesis of GO

Graphene oxide suspension (5 mg/mL) was prepared by the Hummers’ method using commercial graphite powder (Aldrich, <150 μm) as the original material. Firstly, 2 g graphite power, 2 g NaNO_3_ and 96 mL of H_2_SO_4_ (98%) were mixed together at 0°C under stirring. Secondly, 12 g of KMnO_4_ was slowly put in the mixed solution within 30–60 min. Then the mixture was stirred at 0°C for 90 min and at 35°C for 120 min. After that, 80 mL of deionized water was added to the resultant solution drop by drop. After 15 min at 95°C, another 200 mL deionized water was added into the solution. Finally, 10 mL of H_2_O_2_ (30%) was dropped into the mixture slowly to remove the residual KMnO_4__._ The resulting GO suspension was washed repeatedly with deionized water and collected by high-speed centrifugation until the pH was 7. The homogeneous GO suspension was collected after a small amount of black residue was removed by centrifugation at 5000 rpm for 15 min.

### Preparation of SU-8/GO paper

SU-8 photoresist, an epoxy-negative-tone resist product purchased from Microlithography Chemical company (USA) was coupled with GO film as a moisture-inert material layer. Firstly, GO suspension was dropped on the clean glass and dried at room temperature in the air to form GO film. Then, we spin-coated SU-8 on it with a speed of 5000 rpm for 60 s and placed it in the oven under 95°C for 30 min. After that, the entire device was exposed with designed mask using a lithography machine. Continuously, it was kept in the oven for 15 min and a developer was used to remove the unpolymerized SU-8 to construct the expected patterns. Finally, bilayer paper based on SU-8/GO was peeled off from the clean glass. Except for special emphasis, we chose the duty ratio of 1.0 and the period of 400 μm for all of the subsequent experiments.

### Characterization

The SEM images of the samples were characterized by a JEOL JSM-7500 field-emission scanning electron microscope (FE-SEM). The XRD patterns were recorded using a Rigaku D/MAX 2550 diffractometer with Cu Kα radiation (λ = 1.5418 Å). XPS data were measured with an ESCALAB 250 spectrometer. The FTIR spectrum was taken on an FTIR Nicolet 5700 spectrometer. The relative humidity was controlled by using saturated aqueous solutions of MgCl_2_, K_2_CO_3_, NaBr, NaCl, KCl, K_2_SO_4_ and H_2_O in an enclosed container, which generated ∼33%, 44%, 57%, 75%, 86%, 97% and 100% RH, respectively. The relative humidity of our ultra-clean laboratory was measured to be 23%. We switched the RH values by placing the samples inside and outside the closed containers with saturated solution inside. Theoretical simulation was achieved using finite element analysis software.

## Supplementary Material

nwz219_Supplemental_Files
